# Effect of Purified Murine NGF on Isolated Photoreceptors of a Rodent Developing Retinitis Pigmentosa

**DOI:** 10.1371/journal.pone.0124810

**Published:** 2015-04-21

**Authors:** Maria Luisa Rocco, Bijorn Omar Balzamino, Pamela Petrocchi Passeri, Alessandra Micera, Luigi Aloe

**Affiliations:** 1 Institute of Cell Biology and Neurobiology, CNR, Rome, Italy; 2 IRCCS-G.B. Bietti Foundation, Rome, Italy; 3 Dept. Medicine of Systems, University of Rome Tor Vergata, Rome, Italy; Dalhousie University, CANADA

## Abstract

A number of different studies have shown that neurotrophins, including nerve growth factor (NGF) support the survival of retinal ganglion neurons during a variety if insults. Recently, we have reported that that eye NGF administration can protect also photoreceptor degeneration in a mice and rat with inherited retinitis pigmentosa. However, the evidence that NGF acts directly on photoreceptors and that other retinal cells mediate the NGF effect could not be excluded. In the present study we have isolated retinal cells from rats with inherited retinitis pigmentosa (RP) during the post-natal stage of photoreceptor degenerative. In presence of NGF, these cells are characterized by enhanced expression of NGF-receptors and rhodopsin, the specific marker of photoreceptor and better cell survival, as well as neuritis outgrowth. Together these observations support the hypothesis that NGF that NGF acts directly on photoreceptors survival and prevents photoreceptor degeneration as previously suggested by in vivo studies.

## Introduction

Animal models are widely used for investigating the aetiology of Retinitis Pigmentosa (RP) and ultimately for the developing a therapy for the disease [1]. The first animal model of inherited RP (RP) indicating the major characteristics of the human disease is the Royal College of Surgeon (RCS) rat. The visual pathogenesis of this rodent strain comprises a group of inherited progressive retinal cell dystrophies characterized by rod and cone photoreceptor degeneration, leading to progressive loss of vision [2,3]. This eye pathology affects approximately 1 in 400 in a general population and currently a resolute therapy that can arrest or significantly modify the outcome of the disease is not available [4,5]. Thus, any animals models of RP with similar genetic deficits, either occurring naturally or obtained through transgenic manipulations that can provide further information and allow to identify new mechanisms and hopefully suggest potential therapeutic strategies represent a major aim of numerous expert in this field of ocular disorder. The RCS rat is a well-known genetic animal model of photoreceptor degeneration to investigate this aspect, including the identification of biological mediator involved in mechanism of cell death and cell survival. During last few decades, fields of interest of these experimental approaches, include the use of drugs delivery [6] transplantation of retinal pigment epithelium (RPE) or photoreceptors [7–10], gene therapy [11], the administration of growth factors [12]. The first evidence of possible neuroprotective effect of growth factor on retinal cell degeneration was reported by [12,13] who demonstrated that beta-fibroblast growth factor might be able to protect degenerating photoreceptors in RCS rats [14]. Unfortunately, it was also found that this growth factor triggers retinal neovascularization making unacceptable for further studies and potential human therapy [14]. More recently, it was reported that retina of mice and rats affected with RP are characterized by reduced presence of Nerve Growth Factor (NGF) and NGF-receptors and suggested that exogenous NGF administration might delay and/or protect photoreceptors degeneration [15–18]. Other studies supporting the hypothesis that NGF is involved in the protection of retinal cells were provided by Siliprandi et al in rat [19] Carmignoto et al in rabbit [20], and in glaucoma by Lambiase and Aloe in rats and humans [21].

Nerve growth factor (NGF) is the first discovered and the best characterized member of the neurotrophin family [22] that includes Brain-Derived Neurotrophic Factor (BDNF) and Neurotrophin-3/4/5 [23,24]. The biological effect of NGF is mediated by two distinct receptors: trkA^NGFR^ (a tyrosine kinase receptors) and p75^NTR^ and the biological activity on target cells depends on their surface trkA^NGFR^/p75^NTR^ ratio [25,26]. Intracerebral administration of purified NGF has been shown to protect basal forebrain cholinergic neurons that are known to degenerate in brain aging and memory loss in laboratory animals [27,28] and in Alzheimer’s disease [29]. The neuroprotective role of NGF was furthermore suggested by our studies showing that in vivo NGF administration protects degenerating retinal ganglion cells and photoreceptors degeneration [18,21,30] that lead the hypothesis that exogenous intra-vitreal or topical NGF administration might be able to rescue degenerating retinal cells. However, clear evidence that NGF acts directly on degenerating photoreceptors is still not available. Whether photoreceptors of rats developing RP express trkA^NGFR^ and NGF action is directed or mediated by local retinal cells have been not yet established, remaining an open question. The herein described in “*in vitro”* experiments were designed to investigate the direct role of NGF on isolated and cultured photoreceptors.

## Materials and Methods

### Ethics Statement and Animals

A total of 96 RCS animals at postnatal day 10 (p10) were housed at the CNR animal facility and handled according to the experimental procedure approved by the Ethical Commission on animal experimentation of the National Research Council (CNR, Rome). All experiments were conducted in accordance with the guidelines for the use of animals stated by the Association for Research in Vision and Ophthalmic Research. All efforts were made to reduce the total number of animals and to minimize animal suffering. At the time of sampling, animals were sacrificed by means of an overdose of anaesthetic (Ketamine-xylazine and tiletamine-zolazepam; Fort Dodge Veterinaria, S.A., Vall de Bianya-Girona, Spain).

### Chemicals

#### NGF and neutralizing anti-NGF antibodies

NGF was purified in our laboratory from adult male mouse submaxillary glands, following the slightly modified method originally described by Bocchini and Angeletti in 1969 [31]. The anti-NGF antibody (ANA) was raised in goat and purified following the procedure indicated by Stoeckel and coworkers [32].

#### Antibodies

Anti-Rhodopsin (MS-1233-P; Ab-1 (RET-P1); Neomarkers, Fremont, CA, USA); anti-trkA^NGFR^ (sc-118; Santa Cruz Biotech, Santa Cruz, CA, USA); anti-phospho trkA^NGFR^ (p-trkA^NGFR^; sc-130222; Santa Cruz); anti-p75^NTR^ (sc-6188; Santa Cruz); and anti-GAPDH (sc-365062; Santa Cruz). Alexa Fluor 488-conjugated goat anti-rabbit and Alexa Fluor 594-conjugated goat anti-mouse as well as horseradish peroxidase-conjugated anti-rabbit/mouse specie-specific secondary antibodies (Cell Signaling Technology, Danvers, MA, USA).

### Retina dissection, cell cultures and photoreceptor isolation

Retinas were dissected out from whole eyeball according to a standard procedure of retina peel-off and treated with 0.25% Trypsin-HBSS solution (Sigma-Aldrich, St. Louis, CA, USA) for 10 min to release single cells. Single cells (5,000 cells/well) were seeded on precoated (0.5% poly-Lysine; P2636, Sigma) round-glass coverslips in 24well-plates (Nunc, Roskilde, Denmark) or directly on plates and cultured in DMEM/F12 supplemented with 5% horse serum (respectively from EuroClone and Gibco-Carlsbad, CA, USA) in the presence or absence of 50ng/mL NGF. After 6 days of culturing, cells were checked for live/death ratio according to a standard procedure (Trypan blue vital staining; Flow laboratories, Irvine, Scotland, UK). Cells on coverslips were washed in PBS (0.1M pH 7.5; Applichem, Darmstadt, Germany), briefly fixed in cold 4% buffered paraformaldehyde (5min), quenched in 50mM NH_4_Cl (3min), permeabilized in 0.01% Triton X-100 (5min) and subject to confocal analysis. Other sister cells were trypsin harvested and pellets were subject to Western blot analysis. For bio-molecular studies on isolated photoreceptors, retinal cells from stimulating/neutralizing experiments were harvested, post-fixed in 0.5% cold paraformaldehyde (PFA) and probed with Rhodopsin antibodies. Labeled cells were isolated according to the Tetrameric Antibody Complexes recognizing the Rhodopsin-bearing photoreceptors, as previously reported with minor modifications (EasySep Magnet separation; Stemcell Technologies, Voden Medical Instruments SpA, Milan, Italy) [33]. Photoreceptor purity (~80–90%) was verified by flow cytometry (MACSQuant Analyzer; Miltenyi, GmBH, Germany).

### Biochemical analysis

#### Confocal microscopy

Fixed monolayers were incubated for 18hrs at 4°C with Rhodopsin antibody alone (single-staining; 1:300 diluted) or in combination with trkA^NGFR^ or p75^NTR^ antibodies (double staining; 1:100 diluted). To assess for staining specificity, parallel sections were incubated with purified non-specific rabbit IgG antibodies (isotype; Vector, Burlingame, CA, USA). After appropriate washing, the slides were incubated with Alexa Fluor-488 goat anti-rabbit and/or Alexa Fluor-594 anti-specie-specific IgG secondary antibodies (1:400) for 1hr at room temperature. Nuclear counterstaining was performed with DAPI (0.1mM; Molecular Probes, Eugene, OR, USA). Sections were mounted in home-made anti-fading medium, observed under a confocal microscope (CLSM; SP5 Leica Microsystems; Wetzlar, Germany) and acquired at X60/objective (1024x1024 pixels; 10Hz acquisition speed; 30 or 60 μm pine-hole and low/high laser exposure). No intensity-image adjustment was performed while for presentation purposes color–image changes were performed after assembling in panels (Adobe Photoshop ver.7; Adobe System, San Jose, CA, USA).

#### Western Blot analysis

Pooled retinas (n = 5 pools/6 retinas for each pool) and untreated as well as NGF-exposed retinal cells or photoreceptors were homogenized in loading buffer (0.1M tris-HCl buffer, pH 6.8, containing 0.2M DTT, 4% SDS, 20% glycerol and 0.1% bromophenol blue), separated by 8% or 12% SDS-PAGE (20μL/lane) and transferred (18hrs/4°C) to PVDF membrane (GE Healthcare Bio-Sciences, Pittsburgh, USA). Membranes were incubated for 1hr at room temperature with a blocking buffer (5% non-fat dry milk in TBST: 10mM Tris, pH 7.5, 100mM NaCl and 0.1% Tween-20) and washed three times (10 min each at room temperature) in TBST before incubation (18hrs/4°C) with the following primary antibodies: anti-trkA^NGFR^, anti-p-trkA^NGFR^ or anti-p75^NTR^ (all diluted at 1:1000). Membranes were washed three times (10 min each at room temperature in TBST) and incubated for 1hr with specie-specific horseradish peroxidase-conjugated anti-rabbit/anti-mouse IgG antibodies (1:4000). Specific signal was developed according to the ECL chemiluminescent technique (Millipore Corporation, Billerica, MA, USA) and acquired with the 1D Image Station (Kodak, Milan, Italy). The OD values were quantified with the ImageJ v1.43 software (NIH-http://rsb.info.nih.gov/ij/). For normalization, membrane were stripped and reproved with anti-GAPDH (1:1000). The normalized OD values were used for statistical analysis.

### Molecular analysis

#### Relative real-time PCR

Total RNA was extracted from pooled retinas (n = 5 pools/6 retinas each one) or cultured cells with TRIfast (EuroClone), according to manufacturers’ suggestions and resuspended in 10μL fresh RNase free water (Direct Q5, Millipore Corporation, Billerica, MA, USA). To eliminate any DNA contamination, total RNA samples were treated with RNase-free DNaseI, according to the supplier’s protocol (2U/uL DNAse; AM-1907; Turbo DNA free kit; Ambion Ltd., Huntingdon, Cambridgeshire, UK). Total RNA samples were checked for RNA quantity/purity (>1.8; A280, Nanodrop A1000; Thermo Fisher Scientific Inc., Wilmington, USA) and for absence of RNA degradation (1% agarose gel; SaeKem LE-agarose; Lonza, Milan, Italy). Equivalent amounts of RNA (1μg) were used as template to generate cDNAs in a one-cycler programmable thermocycler (PeqLab Biotech, Erlangen, Germany), according to the IMPROM manufacturer’s procedure (Promega, Madison, USA). The resulting cDNAs were amplified using the SYBR Green PCR core reagent kit (Applied Biosystems, Foster City, CA, USA) in an Opticon2 programmable thermocycler (MJ Research, Watertown, MA, USA), according to a standard procedure. Samples were amplified in duplicate and in parallel with negative controls (either without template or with mRNA as template). Cycle thresholds (Cts) were recorded during linear amplification and normalized to those of referring genes run in parallel (nCts = Ct_target_−Ct_referring_). Averages were calculated from replicates and expressed as normalized Ct or as expression ratio of a normalized target with respect to referring gene expression (absolute fold changes), according to REST analysis. The specific primers and amplification profiles were according to previous studies [34]. Primer specificities were confirmed by single melting curves monitored during amplification and provided at the end of amplification by the Opticon2 software. PCR products were randomly tested for single-band amplification by 2.5% agarose gel separation (SaeKem LE-agarose; Lonza, Basel, Switzerland).

### Statistical analysis

All experiments were performed in duplicate and provided as mean±SEM in the graphics. Unpaired Student t test analysis was performed using the StatView software (Abacus Concepts Inc., Barkley, CA, USA). A p-value ≤ 0.05 was considered significant. The REST/ANOVA-coupled analysis was carried out for molecular comparisons [35].

## Results

### Preliminary observations

Previous studies have shown that in RCS rats, the degeneration of photoreceptors begins at the first post-natal week and continues until the photoreceptor outer nuclear layer disappears completely [16]. Therefore to verify the hypothesis of this study, we selected retinas from 10 day old rats (p10), when a significant number of these cells start respectively to degenerate, lose and die [36]. This photoreceptor reduction has been associated with a decrease of local NGF levels [16].

### NGF biological activity on PC12

**Fig 1:**
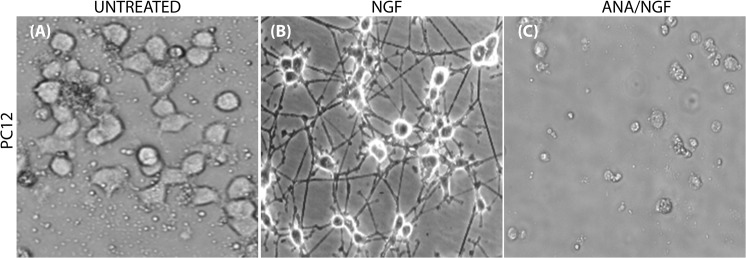
NGF effect on PC12 neuritis outgrew. Phase contrast images confirming the effect of our purified mouse NGF on PC12 cultured for 6 consecutive days alone (A; untreated), with 50ng/ml NGF (B; NGF) and with 50ng/ml NGF pre-incubated with 500ng/ml neutralizing anti-NGF antibodies (C; ANA/NGF). Note the typical neuritis outgrowth from PC12 exposed to NGF (B), as compared to the absence of neuritis outgrowth in untreated (A) and ANA/NGF treated (C) PC12 cultures. (Magnification x200)

First of all, both NGF biological activity and neutralizing anti-NGF antibody (ANA) activities were tested on PC12 cells [37]. PC12 were plated at 10.000 cell density in the presence of medium alone, NGF (50ng/mL) or ANA (500ng/mL). Stimulating/neutralizing activities were determined by evaluating respectively the promoting (50ng/ml NGF) or blocking (50ng/ml NGF pre-incubated with 500 ng/ml ANA) neuritis outgrowth activity on PC12 cultured over 6 days (6d). The results of this preliminary analysis is shown in Fig 1A and 1B respectively.

### The effect of NGF exposure on survival of p10 retinal cells

**Fig 2:**
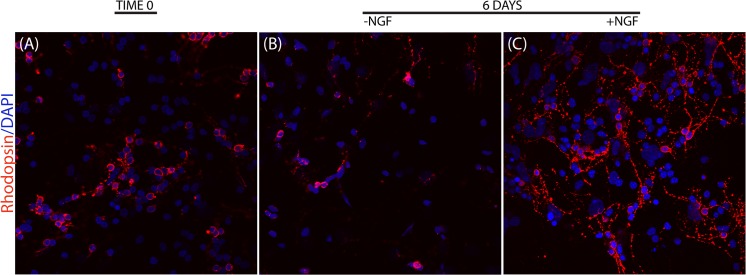
NGF effect on primary cultures of retinal cells. Retinas were dissected out, dissociated and plated according to a standard procedure. Representative confocal images of p10 retinal cells expressing Rhodopsin at baseline (A) or after 6 days of culturing with medium alone (B) or supplemented with 50ng/ml NGF (C). Individual cells were attached as early as 12 hrs from seeding (A). NGF exposed cultures (C) retained retinal cells number, with respect to initial cultures (A) and showed an higher cell number with respect to in untreated ones (B). The red staining is Rhodopsin immunoreactivity in photoreceptors having blue nuclear counterstaining (DAPI). Magnification: x200

Thereafter, we tested the effect of NGF on primary cultures obtained from p10 retinas dissected out, dissociated and plated for 12hrs or 6d depending on the experiment. As shown in Fig 2, the cells quickly adhered to the plate (A, time 0 or 12hrs) and some of those were Rhodopsin positive (red staining). These Rhodopsin positive cells showed few elongations. After 6d of culturing in medium alone, a reduced number of Rhodopsin positive cells was detected, as compared to the initial plating (A). Upon 6d NGF exposure (C), the Rhodopsin positive cell number was comparable to those of plating (A) and interestingly elongations occurred in most of the Rhodopsin positive cells. The effect is clearly visible if comparing NGF treated with untreated fields (C*vs*.B). These observations indicated that NGF stimulates the survival of photoreceptors.

#### The effect of NGF on Rhodopsin positive neuritis outgrowth and trkA^NGFR^ expression

**Fig 3:**
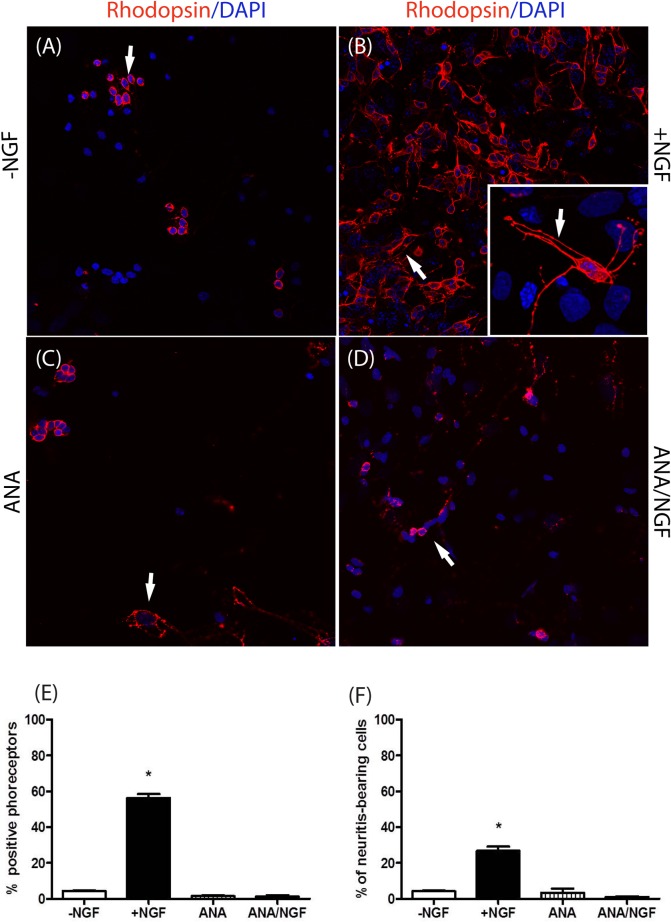
NGF effect on survival and neuritis outgrowth of cultured retinal cells. Representative images of p10 retinal cells cultured alone (A) or in the presence of 50ng/ml NGF (B), 500ng/ml neutralizing anti-NGF antibodies (C; ANA) or with 50ng/ml NGF preincubated with 500ng/ml ANA (D; ANA/NGF) for 6 consecutive days. Note the increased Rhodopsin immunoreactivity in cultures exposure to NGF (B). The Insert (B) is an high-magnification of a Rhodopsin-bearing photoreceptor with neuritis outgrowth. (DAPI nuclear staining; Magnifications: A-D: x400; B (insert): x600/oil immersion). The quantitative analysis indicates that NGF stimulated the survival of photoreceptors (E; averaged cell number) and their neuritis outgrowth (F; length). The results are shown respectively in E and F. Data are expressed as mean±SD per optic field (*p<0.05; NGF-treated *vs*. untreated ones; 3 optic field from 20 untreated and NGF treated wells).

To validate the specificity of NGF effect on these retinal cells, primary cultures were seeded/cultured over 6d in medium alone or supplemented with NGF, neutralizing anti-NGF antibodies (ANA) or NGF pre-incubated with ANA (ANA/NGF). The Fig 3 highlighted the specificity of the addition of exogenous NGF on survival and neuritis outgrowth of dissociated p10 retinal cells, as compared to controls (B *vs*. A). This NGF effect was not detectable in the presence of neutralizing anti-NGF antibodies (C) nor when cells were exposed to NGF pre-incubated with ANA (D). Survival (averaged cell number; E) and neuritis outgrowth (length; F) were quantified in three optic field from 20 untreated and NGF treated wells. The results are shown respectively in E and F (*p<0.05 in the graphics).

**Fig 4:**
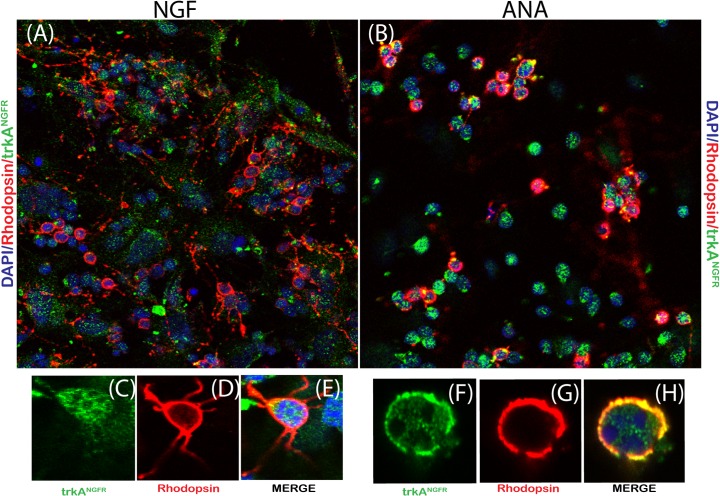
trkA^NGFR^ expression in retinal cells. Representative images of p10 retinal cells exposed to 50ng/mL NGF (A,C-E) or 500ng/ml neutralizing anti-NGF antibodies (B,F-H; ANA) over 6 days. Note the trkA^**NGFR**^ immunoreactivity (green) in Rhodopsin positive (red) cells from cultures exposed to NGF (A) or ANA (B). Single-stainings or merges for NGF (CDE) and ANA (FGH) are shown. (DAPI nuclear staining; Magnifications: A,B: x400; C-H; x600/oil immersion).

According to data illustrated in Figs 2 and 3, these NGF-exposed Rhodopsin positive cells (photoreceptor) survived over 6d of culturing, implying that unless daily supply of NGF is provided, they will degenerate. This hypothesis might be supported by the observation that these cells express trkA^NGFR^. As shown in Fig 4, NGF-exposed cells co-expressed both trkA^NGFR^ and Rhodopsin (A). A widespread neuritis outgrowth and an higher number of trkA^NGFR^ positive cells was observed upon NGF stimulation (B), as compared to untreated or ANA and/or ANA/NGF treated cells (C-D). Indeed, high trkA^NGFR^ and Rhodopsin were co-expressed in NGF treated cultures (green and red staining in single cells in merge panel E; single staining in C-D panels) and co-localized in ANA treated cultures (yellow staining in H merge panel; single staining in F-G panels). Moreover, the confocal analysis indicates that NGF stimulates neuritis outgrowth from photoreceptor.

**Fig 5:**
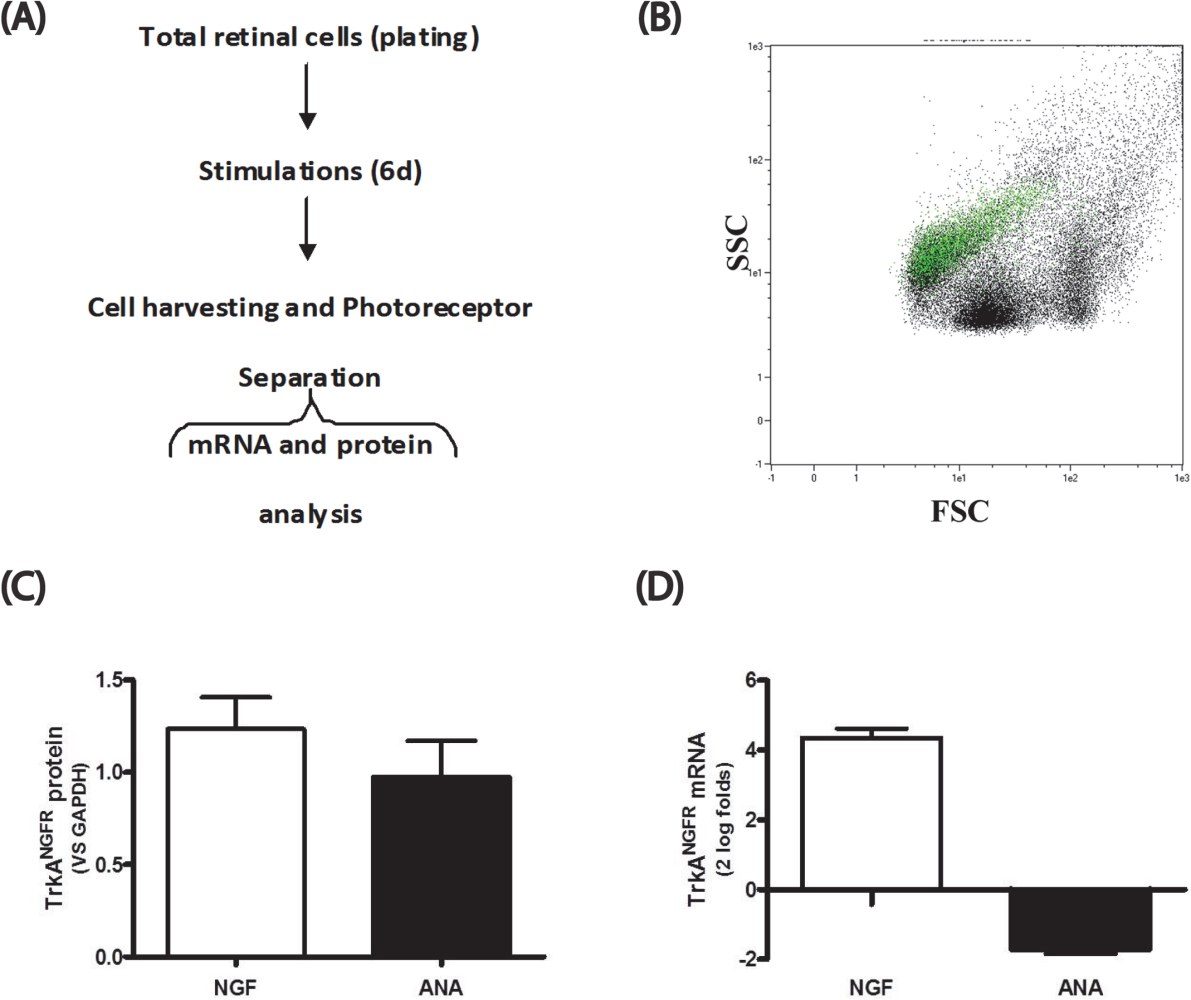
trkA^NGF^ expression in isolated untreated and NGF-treated photoreceptors. Photoreceptors were isolated from p10 retinal cells that were cultured alone (untreated) or stimulated with NGF or neutralized (ANA) for 6 consecutive days. (A) Photoreceptor isolation was performed as described in M&M. (B) Representative side scatter (FSC/SSC) showing the Rhodopsin backgating over a total retinal cell population (10000 events). (C-D) TrkA^**NGFR**^ protein (C) and target gene (D) expression in photoreceptors isolated from cultures exposed to NGF (50ng/mL) or ANA (500ng/mL). Note the increased trkA^**NGFR**^ expression upon NGF exposure. (DAPI nuclear staining; Magnifications: A,B: x400; C-H; x600/oil immersion).

To corroborate the confocal data, retinal cell cultures were cultured in medium alone or supplements and after 6d of culturing, the cells were harvested, fixed and subjected to Rhodopsin-immunobased magnetic separation to provide single Photoreceptor analysis. A flow chart outlining the procedure and a plotter of Rhodopsin-bearing cells backgated on total retinal cells are depicted in Fig 5A and 5B respectively. Isolated photoreceptors (>90% purity) were probed with anti-Rhodopsin antibodies in combination with anti-trkA^NGFR^ specific antibodies. As shown in Fig 5, high trkA^NGFR^ protein (C) and mRNA (D) expression were quantified in photoreceptors isolated from NGF treated cultures, with respect to ANA treated ones (total retinal cells). The results strongly suggested that photoreceptors express trkA^NGFR^ receptor and NGF mediates both survival and neuritis outgrowth effects, as ANA drastically reduced or nearly inhibited (Figs 3, 4 and 5).

#### NGF modulates Bax and Bcl2 expression in p10-isolated photoreceptors

**Fig 6:**
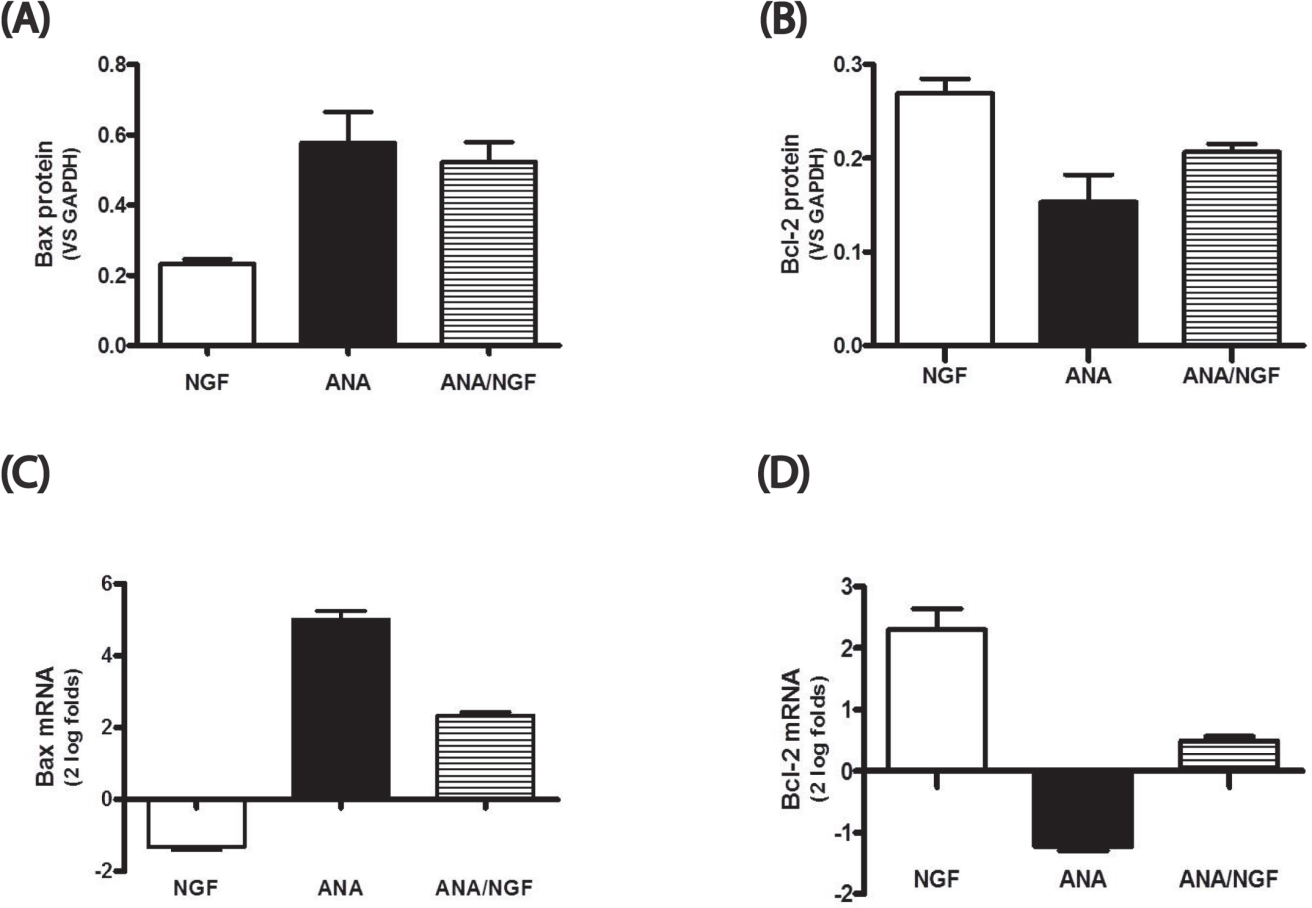
NGF modulates Bax and Bcl2 expression in photoreceptors. Photoreceptors were isolated from p10 retinal cells that were cultured alone (untreated) or stimulated with NGF or neutralized with ANA or ANA/NGF for 6 consecutive days. The histograms show respectively Bax and Bcl2 protein (A-B) and target gene (C-D) expression in photoreceptors isolated from cultures exposed to NGF (50ng/mL), ANA (500ng/mL) and ANA(500ng/mL)/NGF(50ng/mL). Note the increased Bax and decreased Bcl2 expression upon NGF exposure. Similar effects were not observed upon neutralizations (ANA, ANA/NGF). Relative protein levels are expressed as ratio to that of GAPDH. The target gene expression was calculated according to the Rest analysis and expressed as 2log fold-changes with respect to control. *p<0.05

Finally, to provide univocally the NGF effect on photoreceptor survival, Bax and Bcl2 expression were quantified by WB analysis and real time PCR. As shown in Fig 6, a decrease of Bax (A) and an increase of Bcl2 (B) proteins were detected in NGF-treated isolated photoreceptors, as compared to untreated ones (*p<0.05 in the graphics). Corroborating data were obtained with real time PCR, as shown by the decreased expression of Bax (C) and the increased expression of Bcl2 mRNAs (D) (*p<0.05 in the graphics).

## Discussion

The aim of the present study was to investigate whether photoreceptors isolated from retina of RCS rats with RP express NGF-receptors and are affected by direct NGF exposure. Photoreceptors were isolated from ten day-old rats (herein shorten as p10), based on our previous time-course studies showing that photoreceptor degeneration in RCS begin during the second week of post-natal age [16,17]. By the way, whether photoreceptors express NGF-receptors and whether NGF exerts a direct role on photoreceptors, are still open questions.

As a main output of the study, photoreceptors isolated from retina of rats with RP express trkA^NGFR^ and co-express trkA^NGFR^ and rhodopsin as evaluated with immune-localization and western blot analyses. The observation that photoreceptors express both mRNA and protein specific for trkA^NGFR^ is consistent with the hypothesis that NGF exerts a direct action on photoreceptors of rats with RP. The results also revealed that the direct exposure to NGF enhances the expression of both rhodopsin and trkA^NGFR^, implying a potential survival activity [26]. Finally, the fact that these effects are blocked by ANA and NGF/pre-incubated with ANA, that inhibit the biological activity of NGF, indicate that the effect of NGF is strictly dependent by NGF and not due to other factors released by retinal cells. These observations confirmed and extended previous *in vivo* findings that NGF can protect and delay photoreceptor degeneration [16,18].

NGF exposure also stimulated neuritis outgrowth in isolated photoreceptors, as detected by confocal analysis. It is worth mentioning that the neuritis outgrowth promotes neural plasticity by stimulating the formation of new neuronal pathways [38] as well as the neuronal recovery following ischemic/chemical injury within the visual, peripheral and central nervous systems [16,17,28].

Corroborated by previous *in vivo* studies, all the above reported findings support the hypothesis of a protective NGF role in the mechanism of photoreceptor death [17,18].

One critical question raised by this protective action of NGF on photoreceptors is the mechanism through which NGF exerts this protective effect. It has been shown in a consistent number of NGF-target cells that by binding to trkA^NGFR^, NGF up-regulates Bcl-2, a well-known protein exerting a crucial role in protecting cell from apoptosis through a prevention of caspase activation [34,37,39–41]. Our data show that NGF exposed photoreceptors express high levels of Bcl2 and low levels of Bax, supporting the hypothesis that NGF prevents photoreceptor cell death trough anti-apoptotic mechanism(s). The protective effects of the exogenous presence of NGF reinforce this hypothesis. It is therefore possible that the reduced expression of NGF in the retinal photoreceptor layer of rat with RP might be a critical signal that leads progressively to photoreceptor degeneration. To support, it was previously reported that the retina synthesizes and releases NGF and it was hypothesized that exogenous NGF administration can delay and/or protects photoreceptor degeneration [15,16,18]. The herein presented data indicate that NGF acts directly of photoreceptor survival.

To summarize, RP is a group of inherited ocular human disease in which photoreceptor degeneration leads to visual loss and eventually to blindness [1]. A number of findings published over the last two decades suggest that NGF, the first discovered and best characterized neurotrophic factor, is an endogenous neuroprotective molecule and a potential player in the pathogenesis not only for numerous peripheral and brain neurons, but also for visual cells, including damaged corneal cells retinal ganglion cells and photoreceptors [13,42]. NGF takes part in the proliferation, differentiation and functional activity of human, rabbit and rat corneal and retinal cells [27,43–45]. NGF biological activity is mediated by two distinct receptors (trkA^NGFR^ and p75^NTR^) and strictly dependent on their cell surface expression ratio [25,26,46–48]. We previously described that intraocular or topical eye NGF administration can delay and protect retinal ganglion cells from degeneration [19–21,30]. Indeed, we recently reported that intravitreal NGF injection protects RP photoreceptors from degeneration [13,16–18]. Although these *in vivo* results seem to clearly support the hypothesis that NGF acts on degenerating photoreceptors, a clear demonstration of a direct NGF effect has not yet been provided. The present findings on cultures of RP photoreceptors represent the first evidence that NGF acts directly on these cells.

Taken together, these *in vitro* and previous *in vitro*/*in vivo* studies on NGF administration support the hypothesis that the purified NGF molecule represent a critical survival factor not only for RGC degeneration, as observed in Glaucoma and Maculopathy [21,38], but also for photoreceptor degeneration. The availability of human recombinant NGF might encourage pursuing further studies.
